# Interoceptive Accuracy Did Not Affect Moral Decision-Making, but Affect Regret Rating for One’s Moral Choices

**DOI:** 10.3389/fpsyg.2021.746897

**Published:** 2022-02-11

**Authors:** Kaho Tamura, Yoshinari Kobayashi, Hideki Ohira

**Affiliations:** ^1^Department of Cognitive and Psychological Sciences, Graduate School of Informatics, Nagoya University, Nagoya, Japan; ^2^Department of Human Sciences, Toyo Eiwa University, Yokohama, Japan

**Keywords:** moral decision-making, choice, interoception, interoceptive accuracy (IAcc), emotion, regret

## Abstract

Previous studies have revealed the effect of interoceptive accuracy (IAcc), a behavioral measure of the ability to feel physiological states and regulation for that, which origin emotion on decision-making such as gambling. Given that decision-making in moral dilemma situations is affected by emotion, it seems that IAcc also affects moral decision-making. The present study preliminarily investigates whether IAcc affects decision-making and emotional ratings such as regret for one’s own choices in moral dilemma situations. IAcc did not affect moral choice (deontological or utilitarian option), but affected regret ratings for one’s moral choice in portions of dilemma scenarios. Moreover, people with higher IAcc make deontological choices more rapidly than those with lower IAcc in self-related dilemma scenarios. These results suggest that people with higher IAcc feel stronger emotional conflicts about utilitarian choices but weaker conflicts about deontological choices than people with lower IAcc depending on the moral dilemma scenario.

## Introduction

Moral dilemma studies have been conducted in a broad range of areas, such as psychology, neuroscience, philosophy and ethics. These moral dilemma contexts require people to judge whether actions that involve sacrificing a few lives to save many lives are appropriate or to choose one of two actions in certain dilemma situations (Greene et al., 2001). Typically, a moral dilemma context is established in such a way that one option involves a utilitarian emphasis on the number of people saved, such as sacrificing a few lives to save many lives. The other option is deontological and emphasizes justifying the means, such as avoiding sacrificing innocent people. It seems that the decision-making that people face in daily life often has no single correct answer (Nakao et al., 2012); hence, people face dilemmas and feel conflict regarding which choice they should make. Given the nature of decisions that people make in real-life situations, it is important to focus on decision-making in moral dilemma situations.

Moral dilemma studies have mostly investigated which situations make people inclined to judge utilitarian action as acceptable and what type of people are prone to judge this action as acceptable. These studies have revealed a distinct neural basis for utilitarian or deontological judgments depending on the type of scenario (Greene et al., 2004, 2001; Koenigs et al., 2012). The most famous dilemma scenarios are the trolley and footbridge dilemma. Each scenario supposes an uncontrollable and runaway trolley. Specifically, the following situations are assumed: if the trolley goes straight, five workers who work on one roadway beyond the trolley will crash with the trolley and die. In the trolley dilemma, if the direction of the trolley on the roadway changes, the five workers are saved. However, there is one worker on the other changed line. In that situation, one worker will die instead of five workers. There is a switch that changes the direction of the trolley in front of the protagonist, who is required to judge whether to switch the direction of the trolley. On the other hand, in the footbridge dilemma, none of the other roadways switch to the changed direction of the trolley. Five workers can be saved from the uncontrollable trolley in the footbridge dilemma by pushing a man on the footbridge into the roadway, where his body can stop the trolley. In this situation, a protagonist can save five workers; however, one man would die instead (Thomson, 1986). It is well known that people are inclined to judge utilitarian options as acceptable in trolley dilemma, while they are inclined to judge utilitarian options as unacceptable in footbridge dilemma. Greene et al. (2001) classified the former as a personal moral dilemma and the latter as an impersonal moral dilemma. Personal moral dilemmas are defined as follows (Greene et al., 2004): the action causes serious bodily harm to a particular party, and the harm does not result from deflecting an existing threat onto a different party. Differences in the judgments of the two types of moral dilemmas have been explained by dual process theory. In impersonal moral dilemmas, the harming action regarding utilitarian attitude elicits little negative emotion; therefore, people judge utilitarian options as acceptable. In personal moral dilemmas, on the other hand, the harming action elicits strongly negative emotions, and many people judge utilitarian options to be unacceptable (Greene, 2013). In particular, people with emotional insensitivity, such as psychopathy or alexithymia, have been reported to be more prone to judge utilitarian actions as acceptable in personal moral dilemmas (Koenigs et al., 2012; Patil and Silani, 2014). Moreover, patients with ventromedial prefrontal cortex (VMPFC) deficits, which relate to physiological regulation and the representation of value (Hänsel and von Känel, 2008; Padoa-Schioppa, 2011), are also prone to judge utilitarian actions as acceptable in personal moral dilemmas, accompanied by a decrease in physiological arousal characterized by low-amplitude skin conductance responses (SCRs) (Moretto et al., 2009). In contrast, the amplitude of SCRs before healthy participants conduct moral judgments correlates negatively with the number of utilitarian judgments (Moretto et al., 2009). In other words, people with higher SCRs make fewer utilitarian choices. These results suggest that sensitivity to emotion arising from physiological arousal makes people prone to deontological judgments as a result of refraining from utilitarian judgments in personal moral dilemmas and that insensitivity to emotion makes people prone to utilitarian judgments without emotion-based aversion regarding utilitarian action.

Previous studies have mainly focused on the moral judgment of participants as bystanders. Unlike these studies, we are especially concerned with moral decision-making rather than judgment in moral dilemma situations because people are required to make not only judgments but also decisions, which influence later life. Therefore, it is important to investigate the effect of emotion on moral decision-making as well as moral judgments in moral dilemma situations. However, moral dilemma studies that examine decision-making are still lacking compared to those on judgment.

Interoception is the process by which organisms integrate, sense, and interpret signals originating from within the body (Chen et al., 2021). In particular, interoceptive accuracy (IAcc) reflects performance on objective behavioral measures (Garfinkel et al., 2015), it brings to individual differences in emotional sensitivity. People with high IAcc, as measured by heartbeat counting task (Schandry, 1981), experience more intense emotional arousal states in daily life than people with low IAcc (Barrett et al., 2004). Schandry (1981) showed the extent to which IAcc correlates with emotional sensitivity, which was measured by questionnaire. In addition, research has investigated the extent to which IAcc scores in people with emotional deficits. People with higher psychopathic traits show lower IAcc than participants with no psychopathic traits (Nentjes et al., 2013). Moreover, people with alexithymia, who have difficulty feeling emotion, also report lower IAcc (Shah et al., 2016). These results suggest that higher IAcc relates to sensitivity and lower IAcc relates to insensitivity to emotional experience. It is plausible that the ability to feel physiological states correlates with emotional experience because these neural bases is the anterior insula in common (Critchley et al., 2004; Zaki et al., 2012; Terasawa et al., 2013). In addition, IAcc affects not only emotional sensitivity but also decision-making. Interoception contributes to the maintenance of homeostasis (Craig, 2009) and leads to better motivational behavior, such as decision-making, to satisfy physiological needs (Gu and FitzGerald, 2014). In light of this, Werner et al. (2009) initially focused on the relationship between interoception and decision-making. Their study showed that people with high IAcc less frequently choose disadvantageous options than those with low IAcc in the Iowa gambling task (IGT). Moreover, individuals with higher IAcc show greater aversion to monetary-reward loss than individuals with lower IAcc in decision-making task with risk (Sokol-Hessner et al., 2015). These results suggest that the IAcc-based intensity of emotional experience affects emotion-based decision-making.

Given the relation between IAcc and emotion-based decision-making, it is likely that IAcc also affects decision-making in moral dilemma situations. The relationship between IAcc and social decision-making, which is deeply related to morality, has been investigated (Lenggenhager et al., 2013; Piech et al., 2017); however, few studies have investigated the direct link between the IAcc and moral decision-making in dilemma situations such as trolley problem. The present study thus aims to examine whether and to what extent IAcc affects moral decision-making in dilemma situations. First, we predict that people with higher IAcc are prone to make deontological choices in personal moral dilemmas. In light of dual process theory in moral dilemmas (Greene and Haidt, 2002), people with lower IAcc, who might be insensitive to negative emotions in response to sacrificing innocent people, can choose utilitarian options without hesitation because they feel little conflict between alternatives, particularly in personal moral dilemmas. On the other hand, people with higher IAcc who are sensitive to emotions might feel strong negative emotions in response to utilitarian action and be averse to taking such actions in personal moral dilemmas. In addition, we predict that the IAcc level correlates with the speed of moral decision-making in personal moral dilemmas. When people with higher IAcc make utilitarian choices in personal moral dilemmas, they would take longer because they feel more conflict and require more effort to cognitively regulate to overcome deontological choices. Hence, IAcc and reaction time would positively correlate in people who make utilitarian choices in personal moral dilemmas.

Decision-making in moral dilemmas, in particular, elicits conflicts between alternatives and negative emotions; thus, a second goal of this study is to investigate the effect of IAcc on the intensity of negative emotion accompanying decision-making. Among the emotions that people feel in daily life, regret is a negative emotion that is deeply related to and sometimes attached to people’s own decision-making (Zeelenberg et al., 2002). In fact, moral dilemma research has confirmed that people report feeling regret in moral choices (Szekely and Miu, 2015). Therefore, we measure regret ratings for participants’ own moral choices. However, in imaginary experimental moral dilemma situations, it is difficult for participants to feel actual regret following their own moral decisions. Therefore, we measure forecasting regret for one’s own moral choices as a regret rating. Given that IAcc contributes to the intensity of emotional experiences (Barrett et al., 2004), we also predict that people with higher IAcc regret their harmful actions resulting from their utilitarian choices more intensely than those with lower IAcc.

## Materials and Methods

### Participants

Thirty-three female undergraduate students participated in this study. Their ages ranged from 18 to 23 (Mean = 20.84, SD = 1.39). The participants gave written informed consent to participate in the study and could leave it at any time. They conducted a heartbeat counting task and a moral dilemma task. This study was approved by the ethics committee of Nagoya University. Data from this study were assembled before the COVID-19 pandemic.

### Heartbeat Counting Task

Interoceptive accuracy was assessed by a heartbeat counting task (Schandry, 1981). A sensor (emWave PC system, Heart math company) was attached to each participant’s left earlobe to measure the participant’s actual heartbeats. The participants were asked to concentrate on their internal body states and count their own heartbeats during a period indicated by auditory signals of “start” and “stop.” After the participants heard the stop signal, they reported the number of counted heartbeats. This task contained three trials with varying durations (25, 35, and 45 s), and the participants were not informed of these durations. During the task, they were forbidden to take their pulses or use other manipulations to detect their heartbeats. They were asked to stay as still as possible to avoid confounding data from the experimental devices. Each participant’s IAcc score was calculated by the following formula: 1/3Σ (1-| actual number–reported number| /actual number) (Montoya et al., 1993; Pollatos and Schandry, 2010). In addition, the score was multiplied by 10 to apply logistic regression analysis. The IAcc ranged from 0 to 10, where 0 indicates the least accurate heartbeat counting and 10 indicates the most accurate counting. That is, higher scores indicate higher IAcc.

### Moral Dilemma Task

The six scenarios of the moral dilemma task in the present study were selected from the set of scenarios collected and modified by Christensen et al. (2014), except that one scenario was modified for the purposes of our study (see Supplementary Material). We selected only dilemma scenarios that are feasible in real life and could be used to measure people’s tendency to make moral decisions as Tassy et al. (2013) stated that “an action is obviously what the participants think their action could be if they were to make the decision in real life.” With this point in mind, three personal moral dilemma scenarios and three impersonal moral dilemma scenarios were selected: “Bus plunge,” “Orphanage,” “Crying baby,” “Vaccine test,” “Donation,” and “Trolley.” In addition to personal force, these scenarios involve three conceptual factors (Christensen et al., 2014): “Benefit recipient,” “Evitability,” and “Intentionality.” “Benefit recipient” involves whether the protagonist’s life was at stake in the moral dilemma. Cases in which the protagonist’s life is at stake are “Self-beneficial,” and those in which the other’s life is at stake instead of the protagonist’s life are “Other-beneficial.” “Evitability” involves whether the people to be sacrificed by utilitarian action are destined to die anyway. “Intentionality” involves whether the harm is willed and used instrumentally or as a side effect. These four factors are mixed in moral dilemma scenarios because the dilemma scenarios, by their nature, inevitably involve more than one factor. The factors included in each scenario are shown in Table 1.

**TABLE 1:** Each dilemma scenario included one of the two factors in each category: either self or other in the Benefit recipient category, personal or impersonal in the Personal force category, inevitable or avoidable in the Evitability category, and accidental or instrumental in the Intentionality category.

Scenario	Benefit recipient		Personal force		Evitability		Intentionality	
	Self	Other	Personal	Impersonal	Inevitable	Avoidable	Accidental	Instrumental
Bus plunge	○		○		○		○	
Vaccine test		○	○			○	○	
Orphanage	○			○		○		○
Donation		○		○	○			○
Trolley		○		○		○	○	
Crying baby	○		○			○	○	

The participants sat in front of a monitor. PsychoPy3 software (Peirce et al., 2019) installed on a laptop computer was used to present the stimuli and to record the participants’ responses. In each trial, a moral dilemma scenario was presented on the monitor after a certain resting time. The participants read the scenario and went on to the next page by pressing a key, where questions about the dilemma scenario were presented, and responded to them by pressing keys using their right hands.

**FIGURE 1:**
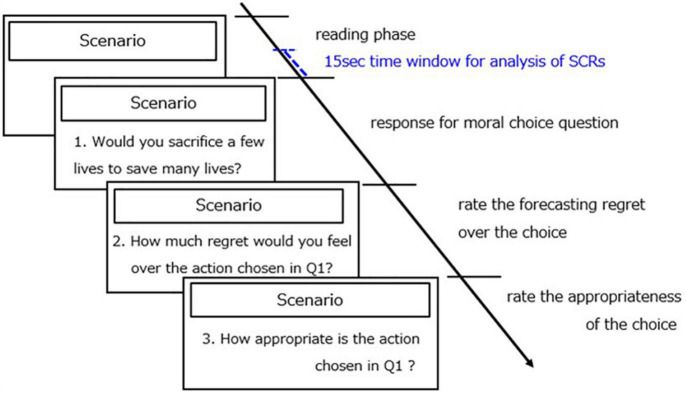
The flow of a moral-dilemma trial. Each trial required the participants to answer three questions about one moral dilemma scenario: The first question asked whether the participants would sacrifice a few lives to save many lives (affirmative answer, utilitarian; negative answer, deontic). The second question asked the participants to rate the extent of the regret they would experience over their answer to the first question. The third question asked the participants to rate the appropriateness of their choice in response to the first question. The participant’s SCRs and reaction time were recorded during the tasks.

Previous studies on moral dilemmas have mainly focused on making judgments about the acceptability or appropriateness of an action as a bystander rather than making decisions. In contrast, the present study was especially concerned with binary decision-making (i.e., deontological or utilitarian choices) of whether to take an action rather than the appropriateness of an observed action, as has been conventionally investigated. Hence, in our experiment, the first question asked whether participants would sacrifice a few lives to save many lives. An affirmative answer to this question corresponds to the utilitarian choice, whereas a negative answer corresponds to the deontological choice. The second question asked how much they would regret if they acted in accordance with the answer to the first question. The participants were asked to report the extent of the forecasting regret they would experience on a seven-point scale ranging from 1 (=no regret at all) to 7 (=regret to an extreme degree). The third question asked to what extent their own choice was appropriate. The participants were asked to report the extent to which their chosen action was appropriate on a seven-point scale ranging from 1 (=not appropriate at all) to 7 (=completely appropriate). The dilemma scenario was presented until the participants answered the third question. The flow of each trial is shown in Figure 1. There was no time limitation because the reaction time was measured. The participants were instructed to read a scenario and choose answers to the questions at their own pace.

To measure the participants’ SCRs while they performed the task, Ag-AgCl electrodes (Vitrode F-150S, Nihon Kohden, Japan) were attached to the middle and ring fingers of their left hands. SCRs were recorded using a unit (DA-3, Vega systems, Japan). After AD conversion, the SCR waveforms were displayed on a laptop computer screen and recorded. These data were analyzed off-line at 10 Hz. For data analysis, we used SCR data that showed the maximum value in the time window between the end of the reading period and 15 s before the period (see Figure 1).

### Statistical Analyses

Regression analysis was conducted to examine the effect of IAcc on the number of deontological choices in all trials. Subsequently, six scenarios were individually analyzed in detail because each dilemma scenario involved different situational factors. First, logistic regression analyses were performed to assess the effects of IAcc and SCR on moral choice, i.e., deontological or utilitarian. Second, Spearman’s rank correlation coefficients were calculated between IAcc and the reaction time for the first question, that is, the time from the end of the scenario reading phase to the time at which a deontological or utilitarian choice was made, to assess whether IAcc is related to the speed of emotion-based intuitive decision-making. Third, hierarchical multiple regression analyses were performed to reveal the effects of IAcc and moral choice on the regret rating. The deontological choice was coded as 0, and the utilitarian choice was coded as 1. Finally, unpaired *t*-tests were conducted to examine whether the extent to which the participants rated their own choice as acceptable varied with the moral choice content.

## Results

One participant was excluded from the data analysis due to a technical problem. Moreover, SCR data were omitted for two participants due to a different technical problem.

**FIGURE 2:**
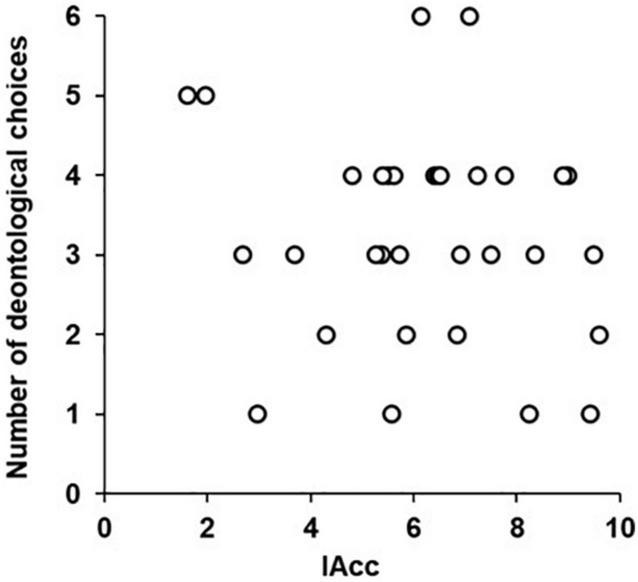
Relation between IAcc and number of deontological choices.

Linear regression analysis was used to reveal the relation between IAcc and the general tendency to make deontological choices. The result showed no significant coefficient (*B* = −0.265, *R*^2^ = 0.027, *p* = 0.369), as shown in Figure 2. Moreover, logistic regression analyses revealed that IAcc and SCR had no significant effect on moral choice for any of the dilemma scenarios. These results are presented in Table 2.

**TABLE 2:** Effect of IAcc and SCR on moral choice.

	Bus plunge	Vaccine test	Orphanage	Donation	Trolley	Crying baby
IAcc	0.013	0.273	−0.142	0.363	0.355	−0.425
SCR	0.103	−0.163	0.003	−0.015	−0.040	−0.454

*This table shows regression coefficient in logistic regression. A value of zero was assigned to deontological choices, and a value of one was assigned to utilitarian choices.*

The results of Spearman’s rank correlation between IAcc and the time until a moral choice was made are shown in Table 3. When the participants made a utilitarian choice in any moral-dilemma scenario, there was no significant correlation between IAcc and reaction time. In contrast, when the participants made a deontological choice, negative correlations were observed between IAcc and reaction time in only Bus plunge (ρ = −0.618, *p* < 0.01), which was reported to induce high arousal state (Christensen et al., 2014).

**TABLE 3:** Rank correlation between IAcc and reaction time for each moral choice.

	Deontology	Utilitarian
Bus plunge	−0.618**	0.371
Vaccine test	0.105	−0.118
Orphanage	−0.186	−0.067
Donation	−0.320	0.091
Trolley	−0.455	−0.027
Crying baby	−0.139	−0.220

**p < 0.01.

**FIGURE 3:**
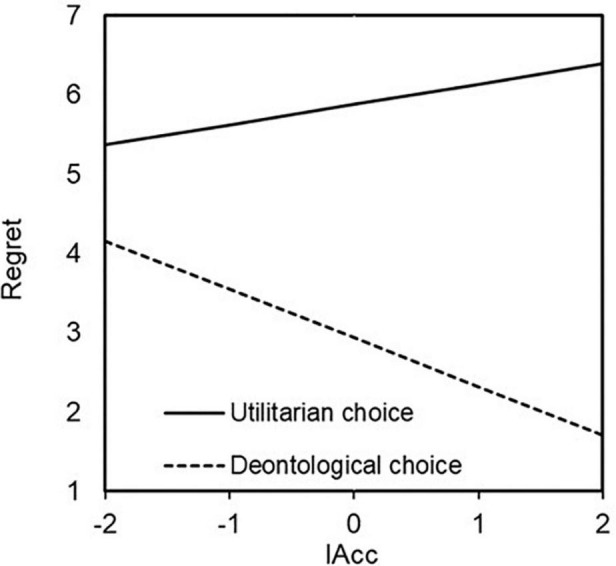
Effect of interaction between IAcc and choice on regret in Orphanage. The higher they rated interoceptive accuracy, the lower they rated regret when they made a deontological choice but not when they made an utilitarian choice (utilitarian choice *B* = 0.257, *n.s*.; deontological choice *B* = –0.613, *p* < 0.000).

**FIGURE 4:**
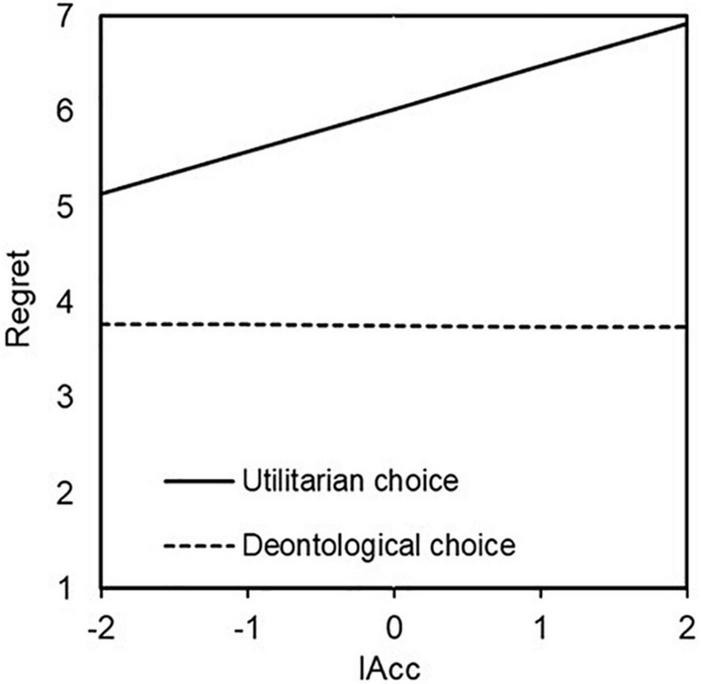
Effect of interaction between IAcc and choice on regret in Bus plunge. The higher they rated interoceptive accuracy, the higher they rated regret when they made a utilitarian choice but not when they made a deontological choice (utilitarian *B* = 0.447, *p* < 0.01; deontology *B* = –0.009, *n.s.*).

Hierarchical multiple regression analyses were conducted to reveal the effects of IAcc and moral choice on regret. In the first step, IAcc and moral choice were included, and in the second step, an interaction term (IAcc and moral choice) was added. A value of 0 was assigned to deontological choices, and a value of one was assigned to utilitarian choices. In five of the six scenarios, there were significant main effects of moral choice (Bus plunge; *B* = 2.272, *p* < 0.000, Vaccine test; *B* = 2.317, *p* < 0.01, Orphanage; *B* = 2.946, *p* < 0.000, Donation; *B* = 2.420, *p* < 0.000, Crying baby; *B* = 1.781, *p* < 0.01). That is, the participants who made a utilitarian choice rated their degree of regret higher than those who made a deontological choice. Moreover, a significant interaction was observed between moral choice and regret in Orphanage. These results are presented in Table 4. Given this interaction, a simple slope analysis of Orphanage was conducted. The participants showed the following tendency when they made a deontological choice in Orphanage: people with higher IAcc, the lower they rated regret. However, the participants showed no such tendency when they made a utilitarian choice in Orphanage (utilitarian choice *B* = 0.257, *n.s.*; deontological choice *B* = −0.613, *p* < 0.000). Simple slope analysis was conducted for Bus plunge as well because there was a weakly significant interaction between IAcc and moral choice (*B* = 0.456, *p* < 0.1). As a result, the participants showed the following tendency when they made a utilitarian choice in Bus plunge: people with higher IAcc rated regret higher. However, the participants showed no such tendency when they made a deontological choice in Bus plunge (utilitarian choice *B* = 0.447, *p* < 0.01; deontological choice *B* = −0.009, *n.s.*). The results of simple slope test are shown in Figures 3, 4.

**TABLE 4:** Effects of IAcc and moral choice on regret.

		Bus plunge			Vaccine test			Orphanage		
		*B*	*R* ^2^	Δ*R^2^*	*B*	*R* ^2^	Δ*R^2^*	*B*	*R* ^2^	Δ*R^2^*
Step1	IAcc	−0.009	0.42***		0.067	0.321**		−0.613***	0.474***	
	Choice	2.272***			2.317**			2.946***		
Step2	IAcc × Choice	0.456^†^	0.484***	0.064^†^	−0.199	0.332**	0.011	0.87***	0.649***	0.175***

		**Donation**			**Trolley**			**Crying baby**		
		***B***	***R* ^2^**	**Δ*R^2^***	***B***	***R* ^2^**	**Δ*R^2^***	***B***	***R* ^2^**	**Δ*R^2^***

Step1	IAcc	−0.317	0.466***		−0.102	0.082		−0.33	0.336**	
	Choice	2.420***			0.901			1.781**		
Step2	IAcc × Choice	0.302	0.494***	0.028	0.162	0.094	0.012	0.394	0.390**	0.054

*In the first step, IAcc and moral choice were included, and in the second step, an interaction term (IAcc and judgment) was added to the hierarchical multiple regression analysis. A value of zero was assigned to deontological choices, and a value of one was assigned to utilitarian choices.*

***p < 0.001, **p < 0.01, ^†^p < 0.1.

The differences in appropriateness ratings for the choices the participants made were assessed by *t*-test and the results are shown in Table 5. The participants who made deontological choices in Crying baby rated their choices as significantly more appropriate than the participants who made utilitarian choices (*t* = 2.938, *p* < 0.01). In the other scenarios, there were no significant differences in the appropriateness ratings of those who made deontological choices and those who made utilitarian choices.

**TABLE 5:** Appropriateness rating by deontological and utilitarian choice for each dilemma scenario.

	Deontology	Utilitarian	
	Mean (SD)	Mean (SD)	*t*-Value
Bus plunge	4.500 (1.319)	4.667 (1.557)	−0.324
Vaccine test	4.619 (1.774)	4.455 (1.527)	0.259
Orphanage	4.667 (1.826)	3.909 (1.514)	1.178
Donation	4.409 (1.869)	4.000 (1.054)	0.644
Trolley	3.818 (1.401)	4.095 (1.375)	−0.528
Crying baby	4.900 (1.524)	3.273 (1.420)	2.938**

**p < 0.01.

## Discussion

The results in the present study, in contrast to our main hypothesis, did not indicated an effect of IAcc on moral decision-making. We address findings for the individual hypotheses.

Our first hypothesis was that people with higher IAcc would be prone to deontological choices in personal moral dilemmas. However, the hypothesis was not confirmed in any moral dilemma scenario. Participants with higher IAcc were not inclined to make deontological choices. First, it has been well confirmed that moral choices are affected by many situational factors of moral dilemmas, participants’ personalities and individual cognitive differences or preferences for the thinking process that they use (Greene et al., 2009; Koenigs et al., 2012; Christensen et al., 2014; Da Silva et al., 2016). The moral choice tendency induced by interoceptive intensity may have been overridden by the moral choice tendency induced by these factors; alternatively, it might be the case that interoceptive intensity alone is too weak to affect moral choices regardless of these factors. Further research is needed to control the influence of these factors on moral decision-making. Second, Awad et al. (2018) reported that there are moral preference differences between people from individualistic cultures and those from collectivistic cultures and people from individualistic cultures showed a preference for utilitarian responses. The present study was conducted with Japanese participants; thus, the participants might have no general preference for utilitarian responses. Because such a bias among the sample might disrupt the effect of IAcc on moral decision-making, further studies should target participants from individualistic cultures, not just collectivistic cultures such as Japan. Third, it remains unclear how interoception and emotions are actually related to each other. Some studies have reported that IAcc is related to self-reported emotional sensitivity or anxiety (Schandry, 1981; Domschke et al., 2010). For example, Schandry (1981) reported that individuals with higher IAcc have higher levels of anxiety and emotional changeability than those with lower IAcc. Domschke et al. (2010) also reviewed the correlation between IAcc and anxiety levels. Considering these studies, it is plausible that the higher IAcc is, the higher emotional sensitivity is. On the other hand, Weiss et al. (2014) reported that IAcc is associated with self-regulation capacity as measured by a self-report questionnaire. Given these findings, people with high IAcc might vary with respect to their levels of emotional sensitivity and cognitive regulation of emotions. If this is the case, high IAcc alone does not determine whether people will make a deontological choice. Some people with high IAcc may have high emotional sensitivity for deontological choices, and other people with high IAcc may have high cognitive regulation ability and be inclined to utilitarian choices. Further studies should consider all the relevant factors behind moral decision-making and examine how each of them contributes to the choices people make.

Moreover, we predicted that when people with higher IAcc make utilitarian choices in personal moral dilemmas, IAcc and reaction time would be positively correlated due to longer cognitive regulation times. However, the tendencies predicted by this hypothesis were not generally observed in the moral dilemma scenarios. On the other hand, a negative relationship between IAcc and choice reaction time for the participants who made deontological choices was found in only one moral dilemma scenario, Bus plunge. This scenario involves many factors that evoke high emotional arousal states (Christensen et al., 2014). Given the extent to which the IAcc correlates with the intensity of the functional connectivity of the saliency network in the posterior insula (Chong et al., 2017), individuals with higher IAcc might easily detect salient stimuli relevant to themselves in self-beneficial moral dilemmas. Thus, for the Bus plunge, it might be that participants with higher IAcc made a deontological choice without hesitation due to promotion-informing somatic sensations serving such as the somatic marker (Damasio, 1994).

Our third hypothesis was that when individuals with higher IAcc make a utilitarian choice in a personal moral dilemma, they would regret it more intensively than individuals with lower IAcc. This hypothesis was not generally confirmed but was partially observed. IAcc was weakly related to the degree of regret in at least two of the three self-beneficial dilemmas. When the participants with higher IAcc made a deontological choice in Bus plunge, they showed low regret; similarly, when the participants with higher IAcc made a utilitarian choice in Orphanage, they showed high regret. These results may suggest that in self-beneficial moral dilemmas rather than personal moral dilemmas, higher IAcc is responsible for the lower regret over deontological choices and the higher regret over utilitarian choices. This suggestion is plausible considering a recent finding: self-beneficial scenarios, where the self is emotionally salient and feels relevant, elicit self-reported high arousal states (Christensen et al., 2014). Moral dilemma situations with a self-beneficial factor may increase emotional arousal; therefore, people with higher IAcc may feel arousal physiological state more strongly than people with lower IAcc (Barrett et al., 2004). As a result, the increase in arousal state may, in turn, trigger an increase in emotions such as regret. If this is correct, it explains why the correlation between IAcc and regret was observed in the self-beneficial dilemma. On the other hand, the relation between IAcc and regret was not observed in all of three self-beneficial dilemmas. Moreover, the dilemma scenarios used in the present study contained factors of various types, and the factors were confounded. In addition, the classification of personal and impersonal dilemmas is controversial because the classification criteria were based on Greene’s intuition and there was no evidence-based clarity criterion (McGuire et al., 2009). Dual process theory was initially proposed to explain the difference in judgment between dilemma situations (Greene et al., 2001, 2004), but the theory does not depend on moral dilemma situations, either personal or impersonal (Greene, 2009). Greene (2009) subsequently mentioned that for both personal and impersonal moral dilemma situations, deontological actions involve emotion and utilitarian action involves cognitive regulation of emotion. Hence, future research should focus on the relation between IAcc and self-beneficial factors in moral dilemma and need to separately investigate possibly influential factors to determine which factors, especially self-beneficial factor, underlies the relation between IAcc and regret ratings in moral dilemma situations.

One unexpected result of our experiments was that when people made a utilitarian choice, they showed high regret regardless of their IAcc. Karneman (2011) noted that people feel intense regret when taking an action against the default. The term “default” here is defined as taking a generally desirable action in a situation. In moral dilemma situations, utilitarian actions involve harming actions that sacrifice a few people’s lives to save many other lives; therefore, it seems that deontological choice is the default, and utilitarian choice is not the default. Hence, the utilitarian choice deviates from the default action and thus might arouse stronger regret than the deontological choice.

The present study investigated forecasted regret accompanying moral choice but did not directly examine the effects of regret avoidance on moral choice. People have tendency to estimate the degrees of regret for each choice option prior to making decisions and to avoid the choices that would arouse greater forecasting regret (Coricelli et al., 2005). Additionally, people have tendency to overestimate post-decisional regret (Gilbert et al., 2004; Sevdalis and Harvey, 2007). It should be useful to examine the effects of such affective forecasting error (Gilbert and Wilson, 2009) on moral decision-making in dilemma situations.

Moreover, significant limitations of IAcc measurement should be mentioned. The present study used Schandry’s heartbeat counting task. This task has been criticized because the heartbeat counting score may be involved in time estimation ability and knowledge of one’s own heartbeat (Desmedt et al., 2018; Zamariola et al., 2018). In addition, given that the task included a low number of trials, the IAcc score in the present study might include a large measurement error. Hence, interpretation related to IAcc in the present study should be carefully conducted. In addition, the present study included only 33 participants. This study suggested the possibility that IAcc affects forecasting regret ratings depending on moral dilemma situations; however, future studies should investigate this effect using larger sample size.

In summary, the present study sheds light on the complex relationships among IAcc, moral decision-making, and the strength of regret for one’s choices. In particular, the results of our experiment showed that people with higher IAcc were not inclined to make a deontological choice in moral dilemma situations. However, they were more likely to feel stronger regret after making a utilitarian choice and weaker regret after making a deontological choice in some self-beneficial dilemmas. These results suggest that people with higher IAcc are inclined to have more negative feelings for utilitarian choices and to face stronger moral conflict in self-beneficial dilemma situations than people with lower IAcc. This study is the first to investigate a link between IAcc and emotion with moral decision-making in dilemma situations.

## Data Availability Statement

The raw data supporting the conclusions of this article will be made available by the authors, without undue reservation.

## Ethics Statement

The studies involving human participants were reviewed and approved by Nagoya University. The participants provided their written informed consent to participate in this study.

## Author Contributions

KT conceived the research idea, provided the experimental design, collected and analyzed the data, and wrote the original draft. YK provided feedback on the experimental design and data analysis. HO provided feedback for the data analysis and supervised the manuscript. All authors contributed to the article and approved the submitted version.

## Conflict of Interest

The authors declare that the research was conducted in the absence of any commercial or financial relationships that could be construed as a potential conflict of interest.

## Publisher’s Note

All claims expressed in this article are solely those of the authors and do not necessarily represent those of their affiliated organizations, or those of the publisher, the editors and the reviewers. Any product that may be evaluated in this article, or claim that may be made by its manufacturer, is not guaranteed or endorsed by the publisher.
