# Supplementary material for: Interoceptive Accuracy Did Not Affect Moral Decision-Making, but Affect Regret Rating for One’s Moral Choices
Source: Front Psychol. 2022 Feb 11;12:746897. doi: 10.3389/fpsyg.2021.746897 (PMC8873090; doi:10.3389/fpsyg.2021.746897)
Supplement: Supplementary file 1 [file Data_Sheet_1.docx]

**SUPPLEMENTARY MATERIAL**

We used the organized sentences of the moral dilemma scenarios from Christensen et al. (2014) in this study. We selected 3rd, 10th, 23th, 29th, 32th, 46th of moral dilemma scenarios and named each scenario based on moral dilemma researches as follow: “Crying baby”,” Orphanage”, “Bus plunge”, “Vaccine test”, “Trolley”, “Donation”.

**Crying baby (3rd)**

Enemy soldiers have taken over your village and will kill all civilians above the age of two. You and ten neighbors are hiding in two rooms of the cellar of a large house. You can hear the voices of soldiers who have come to search the house for valuables. Your baby begins to cry loudly. The crying will attract the attention of the soldiers, who will spare your baby’s life, but will kill you and the other refugees in both rooms.

If you put your hand over its mouth the crying will be absorbed, but your baby will not be able to breathe. This will kill it, but it will save you and the other ten neighbors.

Do you absorb the crying by putting your hand over your baby’s mouth, which will leave it without air, so the soldiers won’t find you and the ten neighbors?

**Orphanage (10th)**

You work in an orphanage in a war swept country that hosts ten orphans. Rebel soldiers have taken over the government and are now killing everyone who does not support their regime. Moreover, they are conducting an ‘ethnic cleansing’ genocide campaign to kill everyone of a particular ethnic group. You and the eleven children of the orphanage did not support the rebellion and you also have one child of this ethnic group in your orphanage. There is no way for you and the other children to escape and the soldiers will certainly kill you all.

If you order a nurse to give the child of the pursued ethnic group a lethal overdose of sleeping pills, the soldiers will believe that you support their regime. This will kill the child, but it will save you and the remaining ten children.

Do you make the soldiers believe you are supporting their regime ordering the nurse to give that one child an overdose, so they will spare your and the other ten children’s life?

**Bus plunge (23th)**

You are the bus driver of a kindergarten trip to the zoo. On the way, faulty tires cause the bus to plunge off a bridge and into a roaring river. You and three of the children are still in the sinking bus. The rest has already been swept away by the river. You grab the two children next to you and begin to swim towards the exit. The third child latches onto your leg. You feel that you are not strong enough to fight against the current and swim with all three children.

If you shake off the third child you will be able to fight against the current, but the child dropped behind will drown. This will kill him, but it will save you and the other two children.

Do you fight against the current shaking off the child, which will make him to drown, so you and the other two children can reach the surface?

**Vaccine test (29th)**

A viral epidemic has spread across the globe killing thousands of people. You are a medical researcher and have developed two substances in your laboratory. Due to a mistake both are labeled as vaccine. One of them is indeed the vaccine, which could save thousands of lives. The other one is lethal.

If you inject the substances to your two lab assistants without knowing which substance is which, you will be able to identify the vaccine. One of the assistants will die, but you will be able to save thousands of lives with your vaccine.

Do you identify the vaccine injecting these two substances into your two lab assistants which will poison one of them, so thousands of people can be vaccinated?

**Trolley (32th)**

A runaway trolley is speeding down the tracks towards five workmen who will be killed if the trolley continues on its present course. You are standing next to the tracks, but you are too far away to warn them. Next to you there is a control switch that can redirect the trolley onto a different track, where only one workman is working.

If you flip the control switch it will divert the trolley onto the track with one workman and will not continue its course towards the five workmen. This will kill this workman, but it will save the other five.

Do you divert the trolley by flipping the control switch, which will cause the trolley to run over one workman, so the trolley does not reach the five workmen?

**Donation (46th)**

You are an electrician hired to fix an electrical problem in the home of a very wealthy tycoon who is terminally ill of cancer. Recently, it has been announced that upon his death, his enormous fortune will be donated to the local children’s hospital. You know that there are 112 children at this hospital who could be saved with this money, but who cannot wait much longer before they die.

(Following sentences were modified for purpose of this study.)

If you short-circuit the cables instead of fixing the problem, the tycoon’s life support equipment will fail, and the essential donation for the children will arrive in time. This will kill the tycoon, but you will save the lives of 112 children.

Do you anticipate the donation by causing the tycoon’s life support equipment to fail, so that the donation gets to the 112 ill children in time?

※ It seemed that the donation dilemma scenario was a mere murder action rather than a dilemma, and it was likely that most people have a bias toward the deontological choice. Therefore, to prevent excessive deontological choice, the second and third paragraphs of the donation dilemma were partially modified, being careful not to change the factors that were included in the original scenario. In general, utilitarian choices in moral-dilemma tasks involve commission acts that sacrifice a few lives to save many lives. Given the omission bias (Spranca, Minsk and Baron, 1991), if we change the utilitarian commission act to a utilitarian omission act, participants might be inclined to choose the utilitarian action. Therefore, we might reduce the bias toward the deontological choice. The modified scenario used in the present study is shown below.

If you neglect to fix the electrical problem, the tycoon’s life support equipment will fail, and the essential donation for the children will arrive in time. This will kill the tycoon, but you will save the lives of 112 children.

Do you anticipate the donation by neglecting the tycoon’s life support equipment so that the donation gets to the 112 ill children in time?

**REFERENCES**

Christensen, J. F., Flexas, A., Calabrese, M., Gut, N. K., Gomila, A. (2014). Moral judgment reloaded: a moral dilemma validation study. *Frontiers in psychology* 5:607. [doi: 10.3389/fpsyg.2014.00607](https://doi.org/10.3389/fpsyg.2014.00607)

Spranca Mark, Miinsk Elisa, Baron Jonathan (1991) Omission and commission in judgment and choice. *Journal of experimental social psychology* 27(1) 76-105. doi: 10.1016/0022-1031(91)90011-T
